# Intra-individual variation of upper airway measurements based on computed tomography

**DOI:** 10.1371/journal.pone.0259739

**Published:** 2021-11-05

**Authors:** Ning Zhou, Jean-Pierre T. F. Ho, Cornelis Klop, Ruud Schreurs, Ludo F. M. Beenen, Ghizlane Aarab, Jan de Lange

**Affiliations:** 1 Department of Oral and Maxillofacial Surgery, Amsterdam UMC and Academic Centre for Dentistry Amsterdam (ACTA), University of Amsterdam and Vrije Universiteit Amsterdam, Amsterdam, The Netherlands; 2 Department of Orofacial Pain and Dysfunction, Academic Centre for Dentistry Amsterdam (ACTA), University of Amsterdam and Vrije Universiteit Amsterdam, Amsterdam, The Netherlands; 3 Department of Oral and Maxillofacial Surgery, Northwest Clinics, Alkmaar, The Netherlands; 4 Department of Oral and Maxillofacial Surgery, Radboud University Medical Centre Nijmegen, Nijmegen, The Netherlands; 5 Department of Radiology, Amsterdam UMC Location AMC, University of Amsterdam, Amsterdam, The Netherlands; Universidade Federal Fluminense, BRAZIL

## Abstract

The aims of this study were (1) to quantify the intra-individual variation in the upper airway measurements on supine computed tomography (CT) scans at two different time points; and (2) to identify the most stable parameters of the upper airway measurements over time. Ten subjects with paired CT datasets (3–6 months interval) were studied, using computer software to segment and measure the upper airway. The minimum cross-sectional area of the total airway and all its segments (velopharynx, oropharynx, tongue base, and epiglottis) generally had the largest variation, while the length of the total airway had the lowest variation. Sphericity was the only parameter that was stable over time (relative difference <15%), both in the total airway and each subregion. There was considerable intra-individual variation in CT measurements of the upper airway, with the same patient instruction protocol for image acquisitions. The length of the total airway, and the sphericity of the total upper airway and each segment were stable over time. Hence, such intra-individual variation should be taken into account when interpreting and comparing upper airway evaluation parameters on CT in order to quantify treatment results or disease progress.

## Introduction

Over the past decades growing awareness of the detrimental effects of obstructive sleep apnea (OSA) has increasingly raised interest in morphometric evaluation of the upper airway [1–3]. Traditionally, upper airway morphology imaging consisted of a two-dimensional (2D) lateral cephalogram [4, 5]. However, due to the technical advancement of computed tomography (CT), this imaging modality has gained increasing popularity [5, 6]. Compared with a 2D lateral cephalogram, CT exhibits the capacity to analyze the upper airway three-dimensionally [7, 8]. Three-dimensional (3D) analysis has been widely used to assess the upper airway, which has given rise to the proposal and usage of multiple methods [3, 9, 10]. Volumetric, areal, and linear measurements, the parameters commonly used for upper airway evaluation, have been shown to have good to excellent inter-operator and intra-operator reliability in previous studies [9–12].

The rationale behind upper airway measurements may be to compare results of an individual to a reference group, or, more likely, to quantify changes within the airway between different time points. While the previous studies quantify the variation and precision of the measurement method itself, measurement on different time points could yield variation in repeated airway measurements as well. It has been proven that the upper airway dimension is influenced by an individual’s body position, head and neck posture, mandibular movement, tongue position, and breathing stage [5, 13–16]. It is a challenge to standardize all these interfering factors during CT scan acquisition [11, 14]. Therefore, even if no airway-influencing intervention has been performed, it is suspected that considerable intra-individual variation in CT volumetric, areal and linear measurements of the upper airway at different time points exists. This variation between time points may hamper adequate evaluation of upper airway changes after surgical and orthodontic procedures, even if a validated measurement method is used. Understanding the degree of intra-individual variation in the upper airway measurements is thus imperative for clinical evaluation and research.

The intra-individual variation of the upper airway measurements has been studied only scarcely. In the study by Obelenis Ryan et al. [11], different volumetric readings of the upper airway were found in the context of different CBCT examinations with identical scanning and patient positioning protocols. However, in their study, the CBCT scans were taken in an upright position. It is well known that upper airway dimension is different between the upright and supine positions [17, 18]. For this reason, a new study under controlled conditions with the patient in supine position during the image acquisition is relevant.

Hence, the primary aim of this study was to quantify the natural intra-individual variation in the upper airway measurements on supine CT scans at two different time points. The secondary aim was to identify the most stable parameters of the upper airway measurements over time, by which accurate evaluation and comparison of the upper airway before and after intervention may be achieved in the future.

## Materials and methods

Due to the retrospective nature of the study and de-identifying patient data prior to conducting the study, the Medical Ethical Committee of the Amsterdam UMC decided that the Medical Research Human Subjects Act was not applicable to this study (Ref. NoW20_261).

### Study population

The population consisted of 10 subjects selected from a patient database of the Department of Oral and Maxillofacial Surgery (5 males and 5 females; mean age 50.3 ± 10.3 years, range 34–68 years), which had two CT datasets (T0 and T1) of the head and neck region acquired in the Amsterdam UMC. They were scanned for various indications, viz., maxillary/mandibular granuloma and palatal fistula, with a 3–6 months’ time interval between scans (mean 4.8 ± 1.2 months). The inclusion criteria were: (1) adequate scan quality; (2) sufficient field of view (sella/nasion to epiglottis base); and (3) time interval between the scans of 3 to 6 months. The exclusion criteria were: (1) patients younger than 18 years; (2) cases with intubation or other potential airway-influencing interventions during or between scans; (3) patients with previous upper airway surgery; and (4) patients with suspected or diagnosed OSA, of whom the upper airway may alter during the progression of OSA disease.

### CT image acquisition

The included spiral CT scans of head and neck were acquired between 2018 and 2019 using the following scanning protocol (SOMATOM Force, Siemens Medical Solutions, Erlangen, Germany): 120 kV, 380 mAs, max. FOV 300 mm, pitch 0.85, slice thickness 1.0 mm, slice increment 1.0 mm, image matrix 512×512, window W1600/L400, hard-tissue kernel H60s. During the imaging procedure, the patients were in supine position and were instructed to remain still with maximum intercuspation, to breathe gently, and not to swallow.

### CT measurements

#### Reference frame

The Digital Imaging and Communications in Medicine (DICOM) files of the CT were imported in Maxilim software (version 2.3.0, Medicim NV, Mechelen, Belgium) for measurements. A hard-tissue reconstruction was created at 300 Hounsfield units (HU) and a soft-tissue reconstruction at -400 HU. To standardize the measurements and minimize the measurement error, the Frankfort Horizontal (FH) plane was constructed for reorientation of the 3D images at T0 [19]. The T1 dataset was superimposed on the T0 dataset, using voxel-based matching on the structures of the cranial base [20, 21].

#### Landmarks

**Fig 1:**
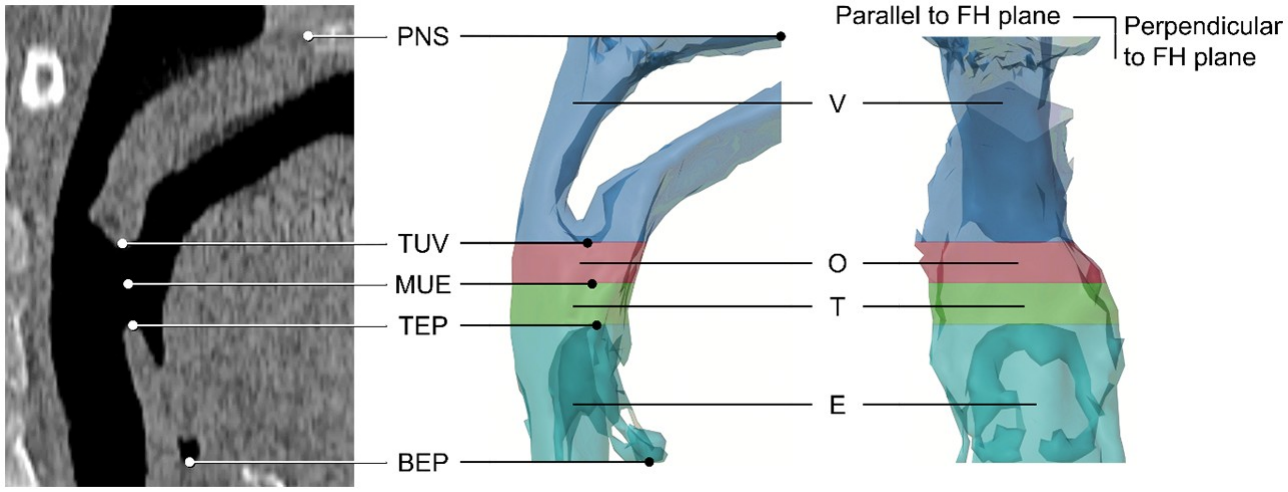
Location of the anatomic landmarks on the midsagittal plane and upper airway subregions of interest defined according to the landmarks. Landmarks: PNS, posterior nasal spine; TUV, tip of the uvula; MUE, midpoint between tip of the uvula and tip of the epiglottis; TEP, tip of the epiglottis; and BEP, base of epiglottis. Subregions: V, velopharynx region; O, oropharynx region; T, tongue base region; and E, epiglottis region.

After re-orientation and superimposition of the paired CT scans, four anatomical landmarks (Fig 1) were identified for segmentation of the regions of interest: posterior nasal spine (PNS), tip of uvula (TUV), tip of epiglottis (TEP), and base of epiglottis (BEP). The reliability of these landmarks has been validated in a previous study [9]. Based on TUV and TEP, the midpoint between them (MUE) was then calculated and localized (Fig 1). Because PNS is a bony landmark and thus an unaltered position between scans, it was localized only once for the T0 scan and re-used for the T1 scan; the other four landmarks were identified on both scans.

#### Boundary

The soft-tissue model was imported into Blender software (version 2.81, Blender Foundation, Amsterdam, The Netherlands) for further analysis. The superior boundary of the upper airway was defined as the plane through the PNS parallel to the FH plane [9, 22]. The inferior boundary was the plane through the BEP parallel to the FH plane [9, 22]. The lateral and posterior boundaries consisted of the pharyngeal walls and the anterior boundary was composed of the soft palate, base of tongue, and anterior wall of the pharynx, with a cut-off at PNS point [10, 23].

#### Segmentation

Based on the identified landmarks, the upper airway was segmented into four distinct regions (Fig 1): velopharynx region (between PNS and TUV), oropharynx region (between TUV and MUE), tongue base region (between MUE and TEP), and epiglottis region (between TEP and BEP). Cutting planes were parallel to the FH plane.

#### Upper airway parameters

**Fig 2:**
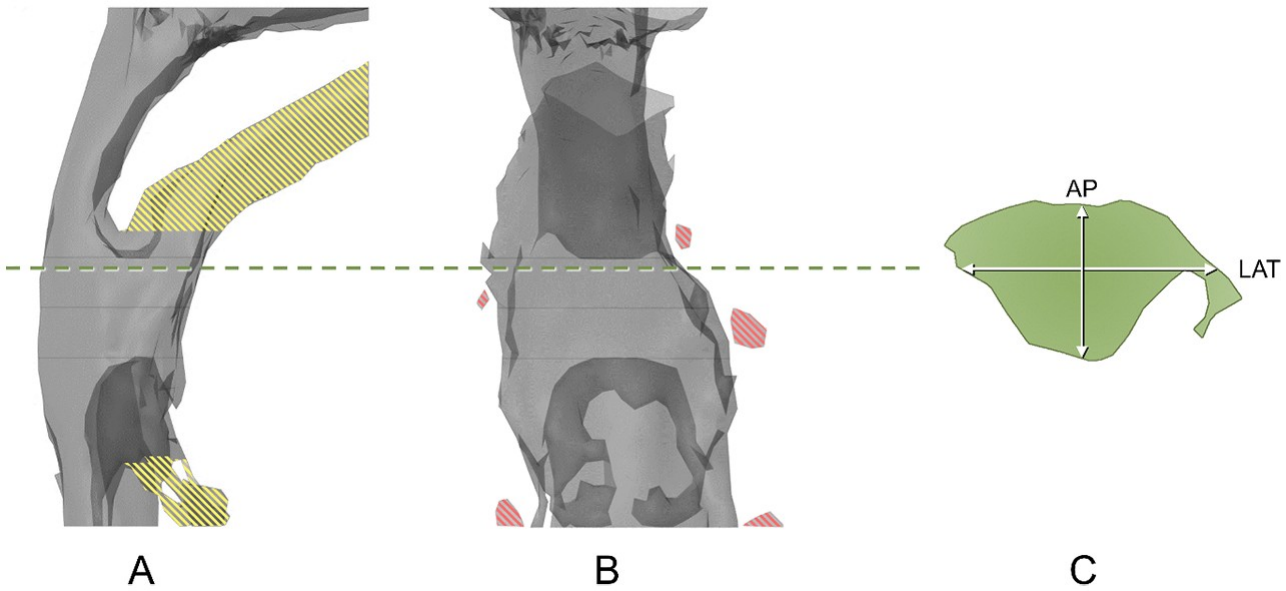
Measurement of minimum cross-sectional area (MCA), using Blender software. (A) “Dead space” (yellow shadow) of the evaluated airway. (B) “Islands” (red shadow) of the evaluated airway. (C) Anteroposterior dimension (AP) and lateral dimension (LAT) of MCA.

One operator (CK), with extensive experience with Blender, performed the measurements in all 20 datasets. The operator was blinded to the measurement results of T0 scans during the measurement for T1 scans. To quantify the inter-operator reliability, a second operator (RS) repeated the entire measurement protocol in five randomly selected datasets. The operators were blinded to each other’s results. The upper airway parameters of interest were volume, length, surface area, minimum cross-sectional area (MCA), and lateral dimension (LAT) and anteroposterior dimension (AP) of the MCA. These parameters were measured for the total airway and for the individual segments (Table 1). Before measuring the MCA, the “islands” (loose air parts) and “dead space” (space in mouth and space between tongue base and epiglottis) were removed from the upper airway model (Fig 2).

**Table 1:** Definition of airway parameters.

Airway parameter	Unit	Definition
**Volume**	mm^3^	Volume of upper airway
**Length**	mm	Length perpendicular to Frankfort Horizontal (FH) plane of upper airway
**Surface area**	mm^2^	Surface area of upper airway without the top and bottom
**Minimum cross-sectional area (MCA)**	mm^2^	At axial view, the minimum cross-sectional area of upper airway after removal of “islands” and “dead space”
**Lateral dimension of MCA (LAT of MCA)**	mm	At MCA, the maximum lateral dimension in an orientation perpendicular to the midsagittal plane
**Anteroposterior dimension of MCA (AP of MCA)**	mm	At MCA, the anteroposterior dimension on the midsagittal plane
**Mean cross-sectional area (meanCSA)**	mm^2^	Equal to the ratio of volume to length (V/L)
**LAT/AP of MCA**	dimensionless (ratio)	Ratio of LAT to AP of MCA
**Airway uniformity**	dimensionless (ratio)	Uniformity of upper airway, equal to the ratio of MCA to meanCSA (MCA/meanCSA)
**Airway sphericity**	dimensionless (ratio)	Mathematical measure of sphericity (how round an object is). A flat object has a sphericity of 0, and a sphere has a sphericity of 1^9^.
		Sphericity = [π^1/3^(6 × V)^2/3^]/SA’
		Note: the closed surface area with top and bottom (SA’) was used for calculating the airway sphericity.

#### Derived airway parameters

Based on these parameters, the following derived parameters were calculated: mean cross-sectional area (meanCSA) [24] for the size of the total airway and each segment; LAT/AP ratio in MCA, airway uniformity [10], and sphericity [10] for the shape of the total airway and of each segment separately (Table 1).

#### Outcome variables

The following outcome variables were derived in this study:

- Intra-individual variation (number of patients = 10; number of CT datasets = 20): the relative difference in the measurements between two scans (T0 and T1) of an individual by operator 1.
- Intra-individual repeatability (number of patients = 10; number of CT datasets = 20): the intra-class correlation coefficient (ICC) for the measurements between two scans (T0 and T1) of an individual by operator 1.
- Inter-operator variation (number of CT datasets = 5): the relative difference between the measurements by operator 1 and operator 2 at T0/T1.
- Inter-operator reliability (number of CT datasets = 5): the ICC for the measurements by operator 1 and operator 2 at T0/T1.
- Agreement and smallest detectable difference (SDD) in the measurements between two scans (T0 and T1) of an individual (number of patients = 10; number of CT datasets = 20) by operator 1.

### Statistical analysis

All data were analyzed using SPSS software (version 26, IBM Corp., Armonk, NY, USA). Descriptive statistical analysis was performed for all demographic and outcome variables.

The intra-individual repeatability and inter-operator reliability of upper airway measurements were evaluated using ICC [25]. Values of ICC less than 0.40, between 0.40 and 0.75, and greater than 0.75 are indicative of poor, fair to good, and excellent reliability, respectively [25]. The relative difference was used to estimate the intra-individual variation and inter-operator variation, which was calculated with the formula: (absolute difference/mean)*100%. Bland-Altman analysis was used to determine the agreement of the airway measurements between two different scans and to obtain the precise confidence interval for paired difference [26]. Based on Bland-Altman’s method, SDD in the airway measurements between two scans of an individual was calculated with the formula: (1.96*SD_T0-T1_).

## Results

Descriptive statistics of all measurements, intra-individual variation estimated by relative difference, intra-individual repeatability estimated by ICC, inter-operator variation estimated by relative difference, and inter-operator reliability estimated by ICC are presented in Table 2. Of the 50 upper airway parameters, the ICC values of intra-individual repeatability were greater than 0.75 for 26, between 0.40 to 0.75 for 19, and less than 0.40 for 5. For the inter-operator reliability estimated by the ICC, all the parameters showed excellent reliability (ICC 0.832–0.999). As for the intra-individual variation in the total airway, the mean relative difference was maximum in MCA (35.5%) and minimum in length (4.9%). Regarding the different airway subregions, the mean relative differences between two scans were exceedingly large (>25%) in: volume at the oropharynx (34.4%), tongue base (29.8%), and epiglottis (25.4%); LAT of MCA at the epiglottis (25.4%); AP of MCA at the velopharynx (28.4%) and tongue base (26.5%); meanCSA at the oropharynx (25.3%), tongue base (26.9%), and epiglottis (25.1%); LAT/AP ratio of MCA at the tongue base (26.3%); and MCA at all levels. The relative differences of the sphericity between two scans in the total airway and each segment were all below 15%.

**Table 2:** Descriptive statistics of variables (N = 10), intra-individual variation and repeatability (N = 10), and inter-operator variation and reliability (n = 5).

	T0		T1		Intra-individual					Inter-operator				
					Variation				ICC	Variation				ICC
	Mean	SD	Mean	SD	Mean	SD	Min	Max		Mean	SD	Min	Max	
**Volume (mm** ^**3**^ **) in the region of**														
**Total airway**	12603.3	5057.8	12455.9	5553.7	21.3	15.8	3.1	52.5	0.836	2.8	2.1	1.4	6.5	0.997
**Velopharynx**	5795.6	2122.3	5720.0	2337.1	15.9	15.0	0.8	47.7	0.913	7.4	4.5	2.8	15.0	0.959
**Oropharynx**	1707.3	1301.1	1679.7	1512.3	34.4	16.4	11.8	65.8	0.895	1.8	1.3	0.1	3.4	0.999
**Tongue base**	1613.3	1089.1	1572.6	1218.2	29.8	17.9	8.5	67.9	0.878	2.7	0.9	1.9	3.9	0.999
**Epiglottis**	3487.1	1650.2	3483.6	1514.9	25.4	29.4	2.6	82.6	0.665	4.9	4.0	1.1	11.0	0.989
**Length (mm) in the region of**														
**Total airway**	66.3	12.4	66.0	12.4	4.9	4.3	0.3	14.1	0.944	1.3	0.9	0.5	2.9	0.997
**Velopharynx**	33.5	6.3	32.4	4.6	6.9	6.2	1.8	18.8	0.850	3.2	2.1	0.7	6.6	0.980
**Oropharynx**	8.4	3.8	8.5	3.9	16.9	13.3	0.0	46.8	0.905	4.8	6.0	0	15.2	0.994
**Tongue base**	8.4	3.8	8.5	3.9	16.9	13.3	0.0	46.8	0.905	4.8	6.0	0	15.2	0.994
**Epiglottis**	16.0	3.0	16.5	2.6	10.2	14.1	2.9	49.4	0.668	4.5	2.3	2.0	8.1	0.949
**Surface area (mm^2^) in the region of**														
**Total airway**	5572.1	1865.1	5512.9	1916.7	16.0	13.2	3.1	36.6	0.829	3.8	3.9	0.2	9.7	0.993
**Velopharynx**	2661.5	695.2	2558.2	730.0	16.6	15.5	0.9	42.0	0.669	5.4	4.1	1.1	12.2	0.968
**Oropharynx**	607.0	471.9	617.5	487.2	22.5	12.6	6.4	47.2	0.966	2.8	2.0	1.3	6.0	0.999
**Tongue base**	525.3	287.6	538.6	303.5	19.0	14.2	2.2	43.4	0.913	3.3	1.7	1.7	5.4	0.998
**Epiglottis**	1778.3	707.0	1798.6	710.0	19.6	14.0	3.4	44.3	0.832	6.9	10.9	0.6	26.3	0.979
**minCSA (mm** ^**2**^ **)in the region of**														
**Total airway**	80.6	54.1	81.1	47.3	35.5	20.3	8.0	69.0	0.845	3.3	7.0	0	16.0	0.980
**Velopharynx**	97.5	75.4	96.6	62.4	30.2	22.4	5.7	69.0	0.912	3.3	7.1	0	16.0	0.980
**Oropharynx**	176.6	83.6	163.8	74.6	31.0	21.2	3.1	73.1	0.740	1.0	1.0	0	2.7	0.999
**Tongue base**	176.0	88.3	156.4	65.6	30.2	18.2	11.2	58.2	0.788	2.9	3.5	0	7.2	0.996
**Epiglottis**	126.0	63.1	108.2	53.6	36.9	26.1	8.0	81.2	0.669	1.4	1.6	0.2	3.6	0.999
**LAT of minCSA (mm) in the region of**														
**Total airway**	15.7	6.7	16.3	7.4	22.4	17.2	2.9	44.2	0.845	1.2	1.9	0	4.5	0.996
**Velopharynx**	16.7	6.3	17.2	6.5	17.6	14.7	5.1	44.2	0.864	2.3	2.8	0	6.1	0.990
**Oropharynx**	21.9	8.2	23.1	7.2	19.1	19.3	0.5	51.6	0.809	3.2	2.9	0	6.9	0.988
**Tongue base**	21.7	6.7	21.2	5.6	12.5	15.4	1.2	49.4	0.822	1.8	1.0	0.6	3.2	0.995
**Epiglottis**	19.7	6.2	18.6	6.1	25.4	22.9	6.2	63.2	0.499	2.3	3.9	0.4	9.3	0.982
**AP of minCSA (mm) in the region of**														
**Total airway**	6.5	3.3	6.1	2.1	27.7	13.3	9.4	51.7	0.781	3.2	4.5	0	10.9	0.929
**Velopharynx**	5.5	2.8	6.0	3.3	28.4	14.0	8.0	52.4	0.852	3.2	4.5	0	10.9	0.960
**Oropharynx**	10.9	2.6	10.4	2.6	16.9	10.3	2.7	30.9	0.713	3.6	3.7	1.4	10.2	0.962
**Tongue base**	11.5	3.1	10.6	2.9	26.5	20.1	3.7	69.4	0.552	3.5	3.2	0	7.6	0.893
**Epiglottis**	8.3	3.2	6.9	2.1	23.2	23.3	5.0	75.0	0.658	6.9	10.3	0	25.2	0.948
**meanCSA (mm** ^**2**^ **) in the region of**														
**Total airway**	194.3	79.5	188.0	69.5	24.4	19.8	2.0	59.4	0.728	2.2	1.5	0.2	3.6	0.998
**Velopharynx**	181.4	72.7	180.5	82.8	22.5	17.5	2.7	59.1	0.861	4.2	3.0	0.3	8.5	0.986
**Oropharynx**	196.0	87.4	181.9	72.3	30.3	21.3	2.4	59.3	0.712	3.0	6.3	0	14.2	0.986
**Tongue base**	193.6	89.4	175.3	61.1	26.9	18.3	1.0	57.0	0.757	3.3	5.7	0.2	13.3	0.975
**Epiglottis**	214.9	91.3	208.8	82.4	25.1	28.0	2.0	86.7	0.605	6.9	10.3	0	25.2	0.997
**LAT/AP of MCA in the region of**														
**Total airway**	2.64	0.85	2.62	0.79	22.7	17.9	2.4	65.4	0.540	4.3	6.3	0	15.3	0.983
**Velopharynx**	3.29	1.08	3.40	2.03	24.2	10.9	12.5	40.9	0.734	5.5	6.5	0	15.3	0.984
**Oropharynx**	2.00	0.56	2.22	0.51	18.3	17.7	0	62.4	0.614	5.4	7.1	0.6	17.0	0.919
**Tongue base**	2.01	0.83	2.18	1.03	26.3	29.5	6.7	94.7	0.126	4.8	3.2	0.6	9.6	0.862
**Epiglottis**	2.46	0.46	2.87	1.09	18.0	18.6	0.5	57.0	0.569	5.2	6.2	0.7	16.0	0.940
**Airway uniformity in the region of**														
**Total airway**	0.41	0.16	0.43	0.15	17.8	15.8	0.1	44.3	0.819	4.8	6.0	0.2	15.3	0.970
**Velopharynx**	0.51	0.20	0.52	0.18	18.9	15.2	0.8	41.6	0.809	5.7	4.3	0.2	10.9	0.978
**Oropharynx**	0.90	0.07	0.89	0.11	8.3	6.4	1.5	19.8	0.533	3.4	5.7	0.1	13.5	0.950
**Tongue base**	0.90	0.06	0.88	0.10	5.0	4.2	0.4	12.2	0.775	5.4	5.0	0.2	13.1	0.869
**Epiglottis**	0.58	0.13	0.52	0.14	21.9	17.1	5.8	59.9	0.372	4.5	4.3	0.2	10.3	0.832
**Sphericity in the region of**														
**Total airway**	0.42	0.04	0.41	0.05	11.1	7.5	2.6	24.9	0.128	3.2	3.6	0	7.9	0.929
**Velopharynx**	0.47	0.05	0.48	0.07	11.4	8.7	1.3	28.8	0.279	3.0	1.8	0.2	4.6	0.945
**Oropharynx**	0.69	0.08	0.68	0.06	7.0	5.1	0.2	16.6	0.633	0.3	0.3	0.1	0.8	0.999
**Tongue base**	0.70	0.06	0.70	0.06	5.0	4.3	0.3	11.1	0.691	0.5	0.3	0.1	0.9	0.991
**Epiglottis**	0.49	0.04	0.49	0.05	8.2	6.5	1.6	18.6	0.304	3.4	5.8	0.1	13.8	0.851

AP, anteroposterior dimension; LAT, lateral dimension; Max, maximum; MCA, minimum cross-sectional area; meanCSA, mean cross-sectional area; Min, minimum; N, number of patients; n; number of CT datasets; SD, standard deviation.

Table 3 shows the results of Bland-Altman analysis of differences between the paired scans (T0-T1; mean, SD, and 95% limits of agreement), as well as the absolute value of differences (|T0-T1|; mean and SD) and SDD values.

**Table 3:** Bland-Altman analysis of difference between two scans (T0 and T1) (N = 10).

	T0-T1		|T0-T1|		95% CI		SDD
	Mean	SD	Mean	SD	Upper	Lower	
**Volume (mm** ^**3**^ **) in the region of**							
**Total airway**	147.5	3037.6	2379.9	1719.9	6101.2	-5806.3	5953.8
**Velopharynx**	75.6	928.9	703.0	565.7	1896.1	-1744.9	1820.5
**Oropharynx**	27.7	645.0	514.0	351.3	1291.9	-1236.6	1264.3
**Tongue base**	40.7	570.3	445.6	326.4	1158.5	-1077.1	1117.8
**Epiglottis**	3.5	1296.8	857.5	929.8	2545.2	-2538.1	2541.7
**Length (mm) in the region of**							
**Total airway**	0.3	4.2	3.1	2.6	8.4	-7.8	8.1
**Velopharynx**	1.2	3.0	2.3	2.2	7.1	-4.8	5.9
**Oropharynx**	-0.1	1.7	1.3	1.0	3.1	-3.4	3.3
**Tongue base**	-0.1	1.7	1.3	1.0	3.1	-3.4	3.3
**Epiglottis**	-0.6	2.3	1.5	1.8	4.0	-5.1	4.5
**Surface area (mm** ^**2**^ **) in the region of**							
**Total airway**	59.2	1105.8	847.9	654.2	2226.7	-2108.3	2167.5
**Velopharynx**	103.3	579.6	415.7	394.6	1239.2	-1032.6	1135.9
**Oropharynx**	-10.5	126.0	111.0	48.0	236.4	-257.4	246.9
**Tongue base**	-13.3	123.0	96.7	70.3	227.8	-254.4	241.1
**Epiglottis**	-20.3	399.4	323.1	209.8	762.5	-803.2	782.8
**MCA (mm** ^**2**^ **) in the region of**							
**Total airway**	-0.6	28.3	24.3	12.1	54.9	-56	55.4
**Velopharynx**	0.9	29.1	23.2	15.8	57.8	-56.1	57.0
**Oropharynx**	12.8	57.1	48.1	29.6	124.8	-99.2	112.0
**Tongue base**	19.6	50.6	45.7	26	118.9	-79.6	99.2
**Epiglottis**	17.8	47.7	40.2	28.8	111.2	-75.7	93.5
**LAT of MCA (mm) in the region of**							
**Total airway**	-0.6	3.9	3.1	2.2	7.1	-8.3	7.7
**Velopharynx**	-0.5	3.3	2.6	1.9	6.0	-7.0	6.5
**Oropharynx**	-1.2	4.8	3.6	3.2	8.2	-10.6	9.4
**Tongue base**	0.5	3.7	2.5	2.7	7.8	-6.7	7.2
**Epiglottis**	1.1	6.2	4.6	4.0	13.2	-11.0	12.1
**AP of MCA (mm) in the region of**							
**Total airway**	0.3	1.8	1.6	0.7	3.9	-3.2	3.6
**Velopharynx**	-0.5	1.7	1.5	0.8	2.8	-3.7	3.3
**Oropharynx**	0.6	2.0	1.7	1.0	4.4	-3.3	3.9
**Tongue base**	0.9	2.8	2.6	1.4	6.5	-4.6	5.6
**Epiglottis**	1.4	2.2	1.8	1.9	5.8	-3.0	4.4
**meanCSA (mm** ^**2**^ **) in the region of**							
**Total airway**	6.3	55.0	41.9	33.5	114.1	-101.5	107.8
**Velopharynx**	0.9	43.5	33.3	25.8	86.3	-84.4	85.3
**Oropharynx**	14.2	60.9	52.3	29.8	133.5	-105.2	119.3
**Tongue base**	18.3	53.4	46.4	28.9	123.0	-86.3	104.7
**Epiglottis**	6.1	77.3	52.1	54.8	157.6	-145.4	151.5
**LAT/AP of MCA in the region of**							
**Total airway**	0.02	0.78	0.60	0.47	1.55	-1.51	1.53
**Velopharynx**	-0.11	1.18	0.87	0.76	2.20	-2.42	2.32
**Oropharynx**	-0.22	0.47	0.38	0.35	0.70	-1.14	0.92
**Tongue base**	-0.17	1.24	0.70	1.01	2.26	-2.60	2.42
**Epiglottis**	-0.41	0.78	0.54	0.68	1.12	-1.94	1.52
**Airway uniformity in the region of**							
**Total airway**	-0.01	0.09	0.07	0.06	0.18	-0.19	0.18
**Velopharynx**	-0.01	0.12	0.09	0.08	0.22	-0.24	0.23
**Oropharynx**	0.01	0.09	0.07	0.05	0.18	-0.16	0.17
**Tongue base**	0.01	0.05	0.04	0.03	0.12	-0.09	0.10
**Epiglottis**	0.07	0.15	0.12	0.10	0.36	-0.23	0.29
**Sphericity in the region of**							
**Total airway**	0.01	0.06	0.05	0.03	0.12	-0.10	0.11
**Velopharynx**	-0.01	0.07	0.06	0.05	0.13	-0.16	0.15
**Oropharynx**	0.01	0.06	0.05	0.03	0.12	-0.11	0.12
**Tongue base**	0	0.05	0.03	0.03	0.09	-0.09	0.09
**Epiglottis**	0	0.06	0.04	0.03	0.11	-0.11	0.11

AP, anteroposterior dimension; CI, confidence interval; LAT, lateral dimension; MCA, minimum cross-sectional area; meanCSA, mean cross-sectional area; N, number of patients; SD, standard deviation; SDD, smallest detectable difference.

## Discussion

This is the first study to evaluate the intra-individual variation of linear, areal, and volumetric measurements of the upper airway in CT scans acquired at two different time points. Because of the short time interval between T0 and T1 (3–6 months), the absence of airway-influencing intervention during or between scans, no airway-influencing pathology or disease present in the patient, and the same position protocol between CT acquisitions, no airway alteration was expected within the scan pairs in our study population. Nevertheless, our findings suggest that different degree of variation exists in each segment of the upper airway between T0 and T1. Although patients with an airway-altering disease (i.e., OSA) or intervention were excluded, this finding may be especially important for evaluating change in these patients as a method to quantify diseases progress or treatment effects.

Regarding the intra-individual variation of the upper airway measurements between T0 and T1 (see Table 2), we found that the MCA of the total airway and of each segment separately generally showed the largest variation, with a relative difference of approximately 30%. Such variation could have two causes. Firstly, the location of MCA is not always constant during the dynamic upper airway movement due to breathing. Secondly, errors or variation in determining the location of the MCA may exist. Although several studies have found that MCA is the most important characteristic of the upper airway that may contribute to distinguishing OSA cases from non-OSA cases [27, 28], caution is thus warranted in interpreting this finding or applying it in clinical practice due to the natural variation found for MCA in the present study.

A significant limitation in CT analysis of the upper airway is differentiating the boundaries of soft tissues and empty spaces (air) by using limited difference in grey levels between them. However, the measurement of upper airway length is not affected by this as it is determined by a user-generated plane. Increased airway length has been suggested to be correlated with the presence and severity of OSA [10]. For consistency and reproducibility, we used a bony landmark having shown excellent reliability in previous studies—PNS—to define the superior boundary of the upper airway [9, 29]. In our study, the length of the total upper airway showed the least variation (relative difference: 4.9%) and it may therefore be regarded as a stable evaluation parameter for the upper airway.

Airway shape may contribute to the development of OSA [1, 10]. Recently, a derived variable, that is sphericity of the upper airway, was suggested and investigated [10, 30]. Klazen et al. found that less sphericity was the main predictor for OSA in patients with craniofacial macrosomia [30]. It is interesting to note that sphericity had low ICC values for intra-individual repeatability; however, it also showed low variation between T0 and T1 in both the total airway and each segment, all the relative differences being below 15%. This may be explained by the fact that ICC is a ratio between inter-unit variability and total variability (intra-unit and inter-unit) [31]. In this study, minor inter-unit variabilities of the sphericity measurements were indicated by the extremely low SDs, which could explain the low ICC values. Therefore, this parameter should not be disregarded based on ICC value alone.

The mean relative differences between two CT scans of the volumes of the total airway, velopharynx, oropharynx, tongue base, and epiglottis were 21.3%, 15.9%, 34.4%, 29.8%, and 25.4%, respectively. Obelenis Ryan et al. [11] evaluated CBCT scans of 27 patients obtained at two time points and reported that the mean relative differences of the volumes of the nasopharynx, oropharynx, and hypopharynx were 9.8%, 17.8%, and 12.0%, respectively. However, care should be taken in comparing the results between the two studies because of the different methodology in the upper airway segmentation. Moreover, differences between CT and CBCT evaluation of the upper airway should be noted. CT are performed when the patient is in the supine position, while most CBCT units acquire images with the patient in the upright position [32]. Soft tissue contrast resolution on CBCT imaging is inferior to CT imaging and therefore segmentation results are different [33].

There are several studies describing the morphometric evaluation of the upper airway [23, 24]. To date, however, there is no methodological standardization in 3D analysis of the upper airway [34]. Chen et al. [9] proposed a method of landmark localization for 3D upper airway measurements, which showed excellent intra- and inter-operator reliability. In the present study, four of the six landmarks proposed by Chen et al. were utilized: PNS, TUV, TEP, and BEP. Additionally, a derived landmark, MUE, was localized at the midpoint between TUV and TEP. Through the experience of over 8,000 drug-induced sleep endoscopy (DISE) examinations, Kezirian et al. [35] found four structures, namely velum, oropharyngeal lateral wall, tongue base, and epiglottis, which play a prominent role in upper airway obstruction. Accordingly, they proposed the VOTE classification system, which has been widely used for characterizing DISE findings. In 3D evaluation of the upper airway, various subregion definitions of the airway have been used in previous studies [11, 22, 23]. However, structure-based assessment for the upper airway cannot be achieved in these methods. Therefore, based on the work by Kezirian et al. [35], the upper airway was divided into four subregions corresponding to the VOTE classification system. Because PNS, TUV, TEP, and BEP demonstrated excellent intra- and inter-operator reliability in the study of Chen et al. [9], the segmentation of the upper airway based on these landmarks may be considered reliable.

In the current study, all the parameters showed excellent inter-operator reliability. Zimmerman et al. conducted a study to assess the reliability of upper airway analysis with CBCT [34]. Interestingly, in contrast to our results, they found that the MCA and total airway volume showed poor inter-operator reliability. It needs to be noted that in Zimmerman et al.’ study, six examiners of varying levels of education and clinical experience separately performed the upper airway analysis, and the reliability improved with the examiner education and experience. In our study, the measurement protocol was conducted by two experienced examiners, which may explain the discrepancy of reliability between the two studies. In addition, unlike their study, we used a fixed threshold for the selection of the upper airway. In this way, the operator’s subjectivity in the threshold sensitivity selection was eliminated. Since it is generally accepted that the inter-operator reliability of the airway measurements is lower than the intra-operator reliability [34], it was decided to evaluate only the inter-operator reliability. Given that the measurement method of the upper airway used in this study is considered to be reliable, it was possible to evaluate the variation of upper airway measurements between repeated CT scans.

For the upper airway analysis, the primary confounding factors during 3D radiographic image acquisition include the individual’s body, head, jaw, and tongue position, as well as the respiratory phase [5, 14, 15]. A systematic review on the effect of head and tongue posture on the dimensions and morphology of the pharyngeal airway concluded that altered head, body, and jaw position had a significant effect on the upper airway dimensions, particularly on the retro-palatal and retro-glossal regions of the oropharynx [14]. In another study by Gurani et al. [5], five sagittal MRI scans from ten subjects in different head and tongue positions were measured. They found that with the head in supine neutral position, the retropalatal, oropharyngeal, and total volumes increased significantly when the tongue was altered from a resting position to the tip of the tongue in contact with the posterior edge of the hard palate (*P* ≤0.05). Schwab et al. [15] investigated the effects of respiration on the upper airway size using cine-CT in 15 normal subjects, 14 snorer/mildly apneic subjects, and 13 patients with OSA, all of whom were scanned in the supine position during awake nasal breathing. In all three groups, there were significant dimensional changes at all anatomic levels of the upper airway during the respiratory cycle, especially in the OSA groups. Therefore, 3D assessment of the upper airway cannot be considered reliable unless all the above confounding factors are controlled during image acquisition. In this study, even with the same patient instruction during CT acquisition, different upper airway readings were found between two repeated CT scans within the same individual, which emphasizes the need for a more standardized patient instruction in terms of posture and breathing phase during image acquisition for evaluation. This needs to be developed and validated in future studies. As recommended by the American Association of Orthodontists White Paper [36], three-dimensional imaging of the airway is a snapshot of a specific moment of the breathing cycle and such technique currently does not represent a proper and reliable risk assessment tool for OSA. The results of the current study reinforce this recommendation.

This study can provide better insight into the real effects of potentially airway-altering procedures on airway size and morphology, such as orthognathic surgery and orthodontics treatment. The differences in the upper airway measurements caused by orthognathic surgery, such as maxillomandibular advancement for OSA treatment, are probably larger than those between two distinct CT scans in our study. However, minor differences in the upper airway measurements should be interpreted cautiously, in particular when quantifying the effect of treatment on the upper airway parameters in a single individual. The SDD provides the amount of potential variation that should be taken into account when interpreting the measurement changes over time at individual level (see Table 3). For example, a SDD of the MCA at the total airway of 61.3 mm^2^ was found in the present study. This suggests that a change in MCA can only be considered to represent a real change if it is larger than 61.3 mm^2^.

Our study has several limitations. First, the sample size might be considered limited. However, it should be mentioned that the sample size is sufficient to demonstrate the considerable intra-individual variation in upper airway measurements. This variation is not expected to decrease with a larger sample size; only its estimate will be more precise [37]. Second, although patients were provided with standardized instructions during CT acquisition, the retrospective nature of the data collection makes it impossible to verify this. While in theory this study could have been performed prospectively, using an enlarged field-of-view, this would have exposed patients who do not need imaging of the complete airway to a larger radiation dose, including vital structures, raising ethical objections to a prospective set-up. This is the reason why we tried to make use of this set of existing radiographic examinations. The fact that most of published studies on 3D evaluation of the upper airway are retrospective studies with various patient instruction protocols, emphasize the difficulty of this issue. Our study highlights that caution should be taken when interpreting the results of upper airway comparison and evaluation using CT, and that a strict protocol is required for repeated measurements and subsequent imaging sessions. Further studies with a larger sample size should be performed to re-determine the natural intra-individual variation of the airway between two CT scans acquired at different time points, using a standardized patient instruction protocol.

## Conclusion

Our study demonstrates that the dimensions and morphology of the upper airway in CT scans can vary considerably within an individual at different time points, even if the same patient instruction protocol for image acquisition is used. The MCA of the total airway and all its segments generally had the largest intra-individual variation, with relative differences of approximately 30%. The length of the total airway had the lowest intra-individual variation, with relative difference of 4.9%. The relative differences of the sphericity between two scans in the total airway and each segment were all below 15%. The length of the total upper airway, and the sphericity of the total airway and each segment were stable over time. Therefore, such intra-individual variation should be considered when interpreting the results of upper airway comparison and evaluation using CT, and the smallest detectable difference is necessary to detect true differences in upper airway measurements over time at individual level.

## Supporting information

S1 File Raw data. (XLSX)
